# Exploring the Association between Sleep Quality and Heart Rate Variability among Female Nurses

**DOI:** 10.3390/ijerph18115551

**Published:** 2021-05-22

**Authors:** Hsiu-Chin Hsu, Hsiu-Fang Lee, Mei-Hsiang Lin

**Affiliations:** 1 Department of Graduate Institute of Health Care, Chang Gung University of Science and Technology, and Department of Internal Medicine, Chang Gung Memorial Hospital, Tao-Yuan 333, Taiwan; hchsu@mail.cgust.edu.tw; 2 Department of Nursing, Chang Gung Memorial Hospital, Tao-Yuan 333, Taiwan; f22066@cgmh.org.tw; 3 School of Nursing, National Taipei University of Nursing and Health Sciences, Taipei 112, Taiwan

**Keywords:** nurses, sleep quality, heart rate variability, autonomic nervous activities, PSQI, HRV

## Abstract

The quality of nurses’ work has a direct effect on patient health, and poor sleep has been positively associated with nurses’ medical errors. The aim of this study was to investigate the relationship between quality of sleep and heart rate variability (HRV) among female nurses. A descriptive cross-sectional correlational study design was used in January 2014 to study female nurses (*n* = 393) employed in a medical center in Taiwan. Data were obtained from several questionnaires. HRV was analyzed with five-minute recordings of heart rate signals obtained using a Heart Rater SA-3000P. Approximately 96% of the participants self-reported a poor quality of sleep. Compared to non-shift nurses, significant decreases were found in total power (TP) and low-frequency HRV among shift-work nurses. However, negative correlations were found between sleep quality and HRV, including total power, low frequency, and the low frequency/high frequency ratio (*r* = −0.425, *p* < 0.05; *r* = −0.269, −0.266, *p* < 0.05). In a stepwise multiple regression analysis, 23.1% of variance in quality of sleep can be explained by TP and heart rate. The sleep quality of female nurses was poor and this affected their autonomic nervous system, which can contribute unfavorable consequences for their health.

## 1. Introduction

Sleep is a complex bio-physiological circadian process that is directly and indirectly related to many daily physical and mental human functions [1,2,3]. Many studies have shown poor subjective sleep quality and deficient stability in objective sleep occurs frequently among nursing staff in hospitals [4]. This situation may negatively impact physical and mental health and the overall work performance of female nurses [5]. According to Akbari et al. [1], poor sleep has been positively associated with nurses’ medical errors, and shift-working nurses are noted as a risk group to suffer from poor quality of sleep. Moreover, some researchers claimed that circadian rhythm sleep disorders, fatigue, and depression are unique risk factors for sleep disturbances, particularly for nurses in a hospital setting [6]. Nurses play a pivotal role on the health care team since they perform a range of safety-critical tasks; the quality of nurses’ work directly affects patient health [1,6,7]. It is worth noting that most (97.81%) of the 205,938 nurses in Taiwan are female [8]. Several researchers have reported that female nurses generally have worse sleep problems than do male nurses [9,10]. Additionally, compared to male healthcare providers, female healthcare providers had higher burnout scores and a greater workload when they attempted to seek a work-life balance [11].

The autonomic nervous system (ANS) plays a vital role in sleep physiology; it regulates cardiovascular function when sleep occurs and controls the transitions to different sleep stages [12]. The relationship between human sleep and autonomic nervous activity is interdependent. Fluctuations in autonomic nervous activity during sleep may play a crucial role in inducing the sleep or wake cycle in each sleep period [13]. Heart rate variability (HRV) is an objective physiological indicator that assesses the activity of autonomous nerves, including sympathetic and parasympathetic activity [14,15]. HRV is a valid, non-invasive procedure that has been widely applied to understand the physiological autonomic changes during different sleep stages. It has also been applied to understand the effect of sleep-disordered breathing, periodic limb movements, and insomnia, both during sleep and wakefulness [12,16,17]. Henelius et al. [18] mentioned that HRV metrics could therefore be exceptionally well suited for measuring sleepiness under normal working conditions.

The power spectrum analysis densitometer analysis was used to observe the distribution of the heartbeat peak spectral power at different frequencies. The Task Force of the European Society of Cardiology and the North American Society of Pacing and Electrophysiology [19] noted that analysis of HRV includes time and frequency domains. The time domains of HRV parameters include standard deviations of all average R-R intervals (SDNN), and higher values indicate a better ability to regulate the ANS and the response to stress; the square root of the mean squared differences of successive R-R intervals is designated as RMSSD. The frequency domains of HRV parameters include high and low frequencies and the high frequency/low frequency ratio. Low frequency (LF) reflects chiefly sympathetic nervous system activity and a fraction of parasympathetic nervous activity, and the higher the value, the stronger the sympathetic activity. The normalized spectral HRV measures low-frequency HRV (LFnu) and high-frequency HRV (HFnu); these values are frequently used in contemporary sleep research studies to quantify modulation of the sympathetic and parasympathetic branches of the ANS [20]. For research purposes the quantities are normalized. Normalization is obtained by dividing the absolute power of each oscillatory component by total power within 0.04 and 0.5 Hz and multiplying by 100 [19]. High frequency (HF) mainly reflects parasympathetic nervous activity, and the higher the value, the stronger the parasympathetic activity. The ratio of low-to-high frequency power (LF/HF ratio) reflects the sympathovagal balance, and the higher the number, the stronger the sympathetic activity. Total power (TP) is the same as SDNN, which can be considered as all the spectra of the frequencies, whereas the variance of the R-R interval values (variance) indicates parasympathetic activity or total activities of the ANS [20,21]. A study by Jarvelin-Pasanen et al. [14] showed that there was a significant difference between the working hours and leisure time in HRV among female nurses during regular and long-term shifts, and the overall HRV of older nurses is lower than that of younger nurses.

Fang et al. [22] found that insomniacs had lower resting high-frequency (HF) HRV than did normal sleepers, but neither the resting low-frequency (LF) HRV nor the LF/HF ratio were different between groups. Additionally, Dettoni et al. [23] found that those with sleep deprivation had statistically increased sympathetic activity (increased LF and decreased HF power). Work activities and sleep time play an important role in producing the maximum and minimum values of the spectral markers LF and HF. Indeed, in every shift, LF and the LF/HF ratio were higher during work and lower during sleep, thus resembling the 24-hour fluctuations of heart rate. In turn, HF was higher during sleeping hours [24]. Therefore, how to mitigate the pathophysiological interference caused by shift work is very important for implementing effective safety and disease prevention strategies [16]. In a study of healthy male shift workers, spectrum analysis based on heart variability within 24 h showed that lower values of LFnu and LF/HF were suggestive of a reduced cardiac sympathetic modulation and were present when compared with observations when performing work tasks at night and when working in the morning as well as evening [24]. At this point, it is possible that HRV parameters can be utilized not only in the measurement of stress or recovery, but also in the evaluation and promotion of occupational health. Among women who worked as registered nurses, a longer duration of rotating night shift work was associated with a small but statistically significant increase in the risk of coronary heart disease [25].

A study on the heart variability of night shift workers found that night shift workers mediated by the circadian rhythm would dull the changes in the autonomic regulation of the cardiovascular system, which could lead to an increased risk of cardiovascular disease among overnight workers [2]. Nurses working on rapid counterclockwise rotating schedules sleep less, suffer more sleep disorders, and have more difficulty maintaining alertness during working hours [26]. In addition, the influence of shift work and sleep disturbances on HRV and autonomic function has been an active area of investigation [27]. Afulani et al. [11] investigated the relationship between HRV and sociodemographic factors among healthcare workers. Afulani’s study found single health providers and those with higher income had higher HRV than did clinical providers who were married and those with lower income, respectively. Therefore, it is essential for health administrator to critically examine the influence of sleep quality among female nurses to provide appropriate strategies that improve their sleep quality and quality of life. However, it remains unclear whether HRV can be used as a biomarker of sleep quality. Therefore, this study aimed to explore the relationship between sleep quality and HRV among female nurses.

## 2. Materials and Methods

### 2.1. Design and Participants

A descriptive cross-sectional correlational study design was implemented. The target population included 1228 registered nurses. G Power3.1 software was employed to estimate the sample size. Owing to diversity in nursing departments (internal medicine, surgery, obstetrics and gynecology, pediatric, emergency room, intensive care unit), the proportional stratified sampling was used, 393 female staff nurses were recruited from a private medical center of northern Taiwan through posters and barcodes. The poster that was posted it in every wards’ conference room clearly provided a description of the research purposes, procedures, and time commitments. Inclusion criteria were: (i) currently working as nurse who had worked for one year or longer, (ii) female registered nurse, (iii) shift work, and (iv) were willing to participate in this study. Directors and supervisors of the nursing departments were excluded from the study. A structured questionnaire was used to collect demographic data as well as data on the factors affecting sleep quality and resting HRV among participating nurses. This study was approved by the Chang Gung Medical Foundation Institutional Review Board research ethics committee (Institutional Review Board approval: 103-5373B, 2 February 2015). All participants were informed about the purpose of the study procedure. Oral and written consent were acquired from all participants.

### 2.2. Measurement Instruments

***Demographic variables:*** This questionnaire included education level, marital status, number of children, shift work information, age, and nursing seniority.

***Sleep quality survey:*** The Chinese version of the Pittsburgh Sleep Quality Index (CPSQI) [28] was used to measure subjects’ sleep quality. The CPSQI is a self-rated questionnaire that assesses sleep quality and disturbances over the past 30 day. The questionnaire identifies seven components of sleep routinely assessed clinically: subjective sleep quality (one item), sleep latency (two items), sleep duration (one item), habitual sleep efficiency (three items), sleep disturbances (nine items), use of sleep medications (one item), and daytime dysfunction (two items). Each of the seven component scores was determined based on scoring guidelines; the scoring of answers was based on a 0 to 3 scale. The sum of the seven component scores yields one global score of subjective sleep quality (range 0 to 21); higher scores represent poorer subjective sleep quality. A global sum of less than 5 indicates good quality, while 5 or greater indicates a poor-quality sleeper. Cronbach’s α values were 0.83 for the original PSQI instrument by Tsai et al. [28]. In the present study, Cronbach’s alpha for this instrument was 0.81.

***HRV measurement:*** Resting HRV was measured in a relaxed sitting position with the Heart Rater SA-3000P (Medi-Core Co., Ltd., Seoul, Korea). A five-minute measurement was taken with a probe attached to the tip of the subject’s left index finger. Data were collected between 9 and 11 AM to account for diurnal fluctuations. Test measurements were taken in a temperature-controlled room (23~26 °C). HRV was automatically analyzed by time domain and frequency domain. The time domain measures included mean heart rate, SDNN, and RMSSD, and the frequency domain measures included LF, HF, total power, and LF/HF ratio. LF and HF were collected as normalized LF and normalized HF. The measurement and physiological interpretation standards of SA-3000P software are in line with the Task Force of the European Society of Cardiology’s and the North American Society of Pacing and Electrophysiology’s [19] calculation process (SA-3000P Clinical Manual ver.3.0). HRV is the interval change between each heartbeat under sinus rhythm. To measure it, we used a medical engineering instrument to take a five-minute smooth RR interval to convert the ECG waveform to a frequency spectrum for data analysis [29]. The spectrum range of the low-frequency band was set to 0.04–0.15 Hz, while the spectrum range of the high-frequency band was 0.15–0.40 Hz. To reduce the impact of HRV monitoring results, first we asked the subject to rest for 10 min in a quiet state, then we took measurements with the Heart Rater SA-3000P for five minutes, and then used the analysis program to calculate the HRV parameters of each subject.

### 2.3. Statistical Analysis

Data were analyzed using SPSS version 20.0 for Windows (SPSS, Inc., Chicago, IL, USA). Descriptive analyses were used to summarize the frequencies, percentages, means, and standard deviations for each of the variables. Students’ *t* tests, and one-way analysis of variance (ANOVA) were conducted to compare the differences of individuals on sleep quality and HRV subscales. To explore the relation between sleep quality and HRV, bivariate Pearson correlation’s product-moment correlation were utilized. Stepwise multiple regression was used to identify predictors of sleep quality. After controlling for the main confounding factor, sleep medications, all significant independent variables were entered to analyze the model. Statistical significance was set at *p* < 0.05 for all statistical tests. Prior to performing the multiple regression, the QQ plot was conducted to test the normality of dependent variable. In addition, when independent variables were categorical variables, a dummy was performed.

### 2.4. Ethical Considerations

This study was approved by the Chang Gung Medical Foundation Institutional Review Board research ethics committee (Institutional Review Board approval: 103–4962B). All participants were informed about the purpose of the study procedure. Oral and written consent were acquired from all participants.

## 3. Results

### 3.1. Characteristics of Studied Group

A total of 393 female nurse participants ranged in age from 20 to 53, with a mean age of 31.48 (SD = 7.41) years, and 75.1% (*n* = 295) of the subjects worked rotation shifts. The subjects had worked as nurses for 1 to 33 years, with a mean of 8.95 ± 6.61 years. The majority (*n* = 376, 95.7%) had earned a college degree. A total of 153 participants (38.9%) were unmarried, and 258 (65.6%) had no children. The mean values of the HRV variables are shown in Table 1.

### 3.2. Subjects’ Sleep Quality

Table 2 presents the results of mean scores of the PSQI global score and its seven components. The overall mean PSQI global score for the sample was 10.20 (SD = 2.85). Minor variations between the mean scores of the seven PSQI subsections were identified. Sleep efficiency varied among the nurses. The majority of the nurses (*n* = 387, 97.5%) had less than 85% sleep efficiency. The results of sleep latency showed that one third of the subjects (34.9%) took 31 to 60 min to fall asleep. The prevalence of self-reported poor sleep quality was found in 377 (95.9%) participants according to the PSQI global scores (≥5). Among the 393 female nurse participants, almost half (51.4%) rated their sleep quality as fairly good, 31% were fairly poor, and 9.2% were very poor. Furthermore, spending more than 60 min falling asleep every night was observed in 74 (18.8%) participants and 96 (24.4%) subjects slept less than five hours per day. Only 2.8% of subjects indicated that they did not have trouble falling asleep again after waking up. A total of 64 respondents (16.3%) reported using prescription sleep medications. The majority of the participants (49%, *n* = 193) complained of daytime dysfunction, such as difficulty in concentrating while working.

### 3.3. Differences between Participants’ Demographic Variables, HRV, and Sleep Quality

As shown in Table 3, comparisons between demographic characteristics, HRV parameters, and PSQI global score showed that significant differences were found between TP (*t* = 4.94, *p* < 0.01) and LF (*t* = 4.10, *p* < 0.01) in non-shift working nurses whose TP and LF were higher than those of shift working nurses. However, educational level, marital status, and number of children were not significantly different in terms of HRV parameters and PSQI global score.

Differences in mean values of HRV parameters between good sleepers and poor sleepers showed that good sleepers had better LF (*t* = −2.36, *p* < 0.05) and TP (*t* = 2.87, *p* < 0.01), corresponding to better sleep quality (Table 4). Food sleepers had better LF, indicating higher sympathetic activation.

### 3.4. Associations between Sleep Quality, HRV Subscales, and Participants’ Demographic Characteristics

There was a statistically significant negative relationship between the TP and PSQI global score (*r* = −0.425, *p* < 0.01). LF correlated significantly and negatively with PSQI global score (*r* = −0.269, *p* < 0.05). Additionally, the LF/HF ratio correlated significantly and negatively with PSQI global score (*r* = −0.266, *p* < 0.05). Age and nursing seniority were correlated significantly and negatively with HF (*r* = −0.244, *p* < 0.05; *r* = −0.377, *p* < 0.01) (Table 5).

### 3.5. Factors Predicting Quality of Sleep

The results showed that TP and heart rate were significant variables for quality of sleep and accounted for 23.1% of total variance (Table 6).

## 4. Discussion

This study explored the relationship between sleep quality and HRV among female nurses. Overall, approximately 96% of participants in this study showed that sleep quality was poor. The findings of this study were inconsistent with the previous studies [30,31]. Tarhan et al. [31] investigated 152 nurses and found that 61.9% of nurses had poor sleep quality. Additionally, Park et al. [30] investigated 188 nurses working in acute hospitals in South Korea, the results showed that the nurses’ prevalence of poor sleep quality was high (79.8%) and pointed out that poor sleep quality may lead to lower nurse productivity. Moreover, the scores on sleep latency, sleep disturbance, and daytime function components in this present study were higher than those in the study by Garcia et al. [32], which investigated 635 registered nurses and found that sleep latency had the greatest mean value. Based on the above statement, the current study reveals a high prevalence of poor sleep quality, sleep latency, sleep disturbance, and daytime function among nurses. A possible explanation for this situation might be attributed to difficulties in the current working conditions, such as lack of nursing staff and excessive workloads. Additionally, there were differences in mean values of HRV parameters between good sleepers and poor sleepers. The present study found that compared to good sleepers, LF decreased and HF increased statistically. This finding confirmed several studies that indicated that the power spectrum analysis of insomniacs usually increases LF, which means increased sympathetic nervous activity, decreased HF, and decreased parasympathetic nervous activity [24,33].

In this study, nurses’ education level, marital status, and number of children were not significantly different in terms of PSQI global score. The findings of the study were congruent with a study by Tarhan et al. [31]. In addition, several studies have reported that many shift workers suffer from sleep disturbance and that shift work is associated with poor sleep quality [31,34]. Sun et al. [35,36] mentioned that sleep problems among shift nurses have been recognized increasingly as a significant issue at both the individual and organizational levels. A recent study investigated the impact of clockwise and counterclockwise rotation of a work schedule on sleep quality. The results of the study indicated that the counterclockwise shift plan is characterized by a high degree of sleep disturbance and a poor balance between work and life [26]. A somewhat different finding emerged in the current study. Notably, the results of the present study showed not significant difference in terms of PSQI global score with shift work. There are two possible explanations for this discrepancy between studies. First, sufficient rest after a night shift may attenuate the sleep disturbance of nurses with rotating schedules. Second, nurses recover better from a previous work shift when working on an ergonomic shift schedule. The results are in line with Sun et al. [35], which reported that adaptation strategies are needed to improve nurses’ sleep quality and prevent sleep loss during frequent shift rotations that cause changes in circadian rhythms when switching between night shifts and day shifts.

A significant difference was also demonstrated between TP (*t* = 4.94, *p* < 0.01) and LF (*t* = 4.10, *p* < 0.01). Non-shift workers had a higher TP and lower LF than did shift workers. This finding was similar to that of some previous studies [4,37,38]. Chung et al. [37] pointed out that long-term night shift nurses were found to have higher LF during non-REM sleep when compared with that HRV parameter in regular shift nurses. Furthermore, consistent with the findings of Shen et al. [4], shift working female nurses had lower TP of HRV. In addition, job strain and low job control were associated with lowered LF power of HRV among resident physicians [38]. Nevertheless, these findings were in contrast to those of several previous studies [34], which reported no differences in circadian phases between shift workers and non-shift workers. Järvelin-Pasanen et al. [14] reported that during work shifts, an increase in sympathetic and a decrease in parasympathetic control of HRV was observed when compared to a leisure-time situation; thus, the rhythm of HRV was affected by the level of physical activity during a work shift. Moreover, Fan et al. [39], who investigated the relationship between sleep quality and the ANS among shift-working nurses found an increase in LF and a decrease in TP of HRV among the shift-working nurses, although the difference was not statistically significant. Shift situation is an important factor impacting on health status. Shift work causes stress, enhances sympathetic nerve activity, and is an established risk factor for cardiovascular and metabolic diseases [6]. The present study found that the shift situation affects the analysis of autonomic balance of heart rhythm variation, but not statistically significantly, except for TP and LF. The possible reasons are age and seniority. The average age of nurses the present study was low, and the average age was 32 years old, which may make the HRV of shift work less significant. This finding was similar with a study by Ito et al. [40] that examined a staff of ten nurses with an average age of 31.48 ± 7.41 years and found that there was no significant difference in the HRV-related index between night shift and day shift. Another study revealed that when younger workers have more shift work, the variability of their heart rhythm is still better than that of the older ones [41]. The reasons for this finding can be explained by the fact that increases in sympathetic activity and reductions in parasympathetic tone have been reported among night workers relative to those working days, or on days off relative to night work [6]. Another possible explanation may have to do with the amount of physical activity engagement during working hours. Khan [6] found that compared with the pre-shift, physical activity during the night shift increased significantly among nursing staff. Based on the results of the above studies, the time needed for rejuvenation of ANS among shift workers is still in dispute and needs further exploration.

A previous study reported that there was a significant relationship between HRV and daytime functioning among insomniacs and normal sleepers, finding that insomniacs had decreased LF of HRV [42]. The findings of the present study were congruent with those of the above study. Compared with poor sleepers in this study, good sleepers showed better LF and TP. However, another study pointed out that poor sleepers had a significantly lower standard deviation of RR intervals (SDNN; a measure of HRV) compared to that of good sleepers [17]. Thus, it was evident that LF and TP were the significant factors affecting sleep quality in this study. In addition, the results of the present study demonstrated that age and nursing seniority were significantly and negatively correlated with daytime dysfunction, TP and HF. This finding confirmed that of several studies that indicated that age and HRV correlated negatively [41,42]. The literature reveals that several factors influence HRV [41]. One study pointed out that HRV analysis is affected by many factors, including caffeine, age, disease, BMI, day and night festivals, heart rate, acute psychological stress, etc. [43]. Moreover, one study reported that rural ambulance paramedic shift workers are at particular risk for increased levels of fatigue and depression (regardless of age or gender) and poor-quality sleep [44]. Therefore, further explorations for factors contributing to quality of sleep among nurses are recommended. Our study showed that the female nurses’ quality of sleep was poor and female nurses on shift work may have a cardiac autonomic imbalance. Since shift work is a necessity in the nursing profession, the findings of this present study may be of value in developing strategies and education programs to reduce sleep-related problems among nurses.

### Limitations

The current study has several limitations. First, the cross-sectional nature of this study limited the assessment of temporality and therefore causality. A longitudinal study with observations of individual changes in HRV is recommended that applies the findings in an occupational health care setting. Second, there were no data from a nonmedical general population as a control group in the study design to compare differences in sleep disturbances between hospital staff nurses and the general population. Third, the shift status of the subjects on the day of or the day prior to the measurement of heart rate variability was not completely consistent (some ended the night shift for the medical examination, while some used the night shift for it or took vacation), so that may have affected the HRV measurement results. Finally, HRV analysis is affected by many factors, one of which includes sex hormones. This present study did not include the menstrual cycle or menopause, which may affect the results.

## 5. Conclusions

The results of this study indicate that the female nurses’ quality of sleep was poor, and it revealed that shift-work female nurses may have a cardiac autonomic imbalance with a possible enhanced parasympathetic modulation, as assessed by short-term frequency domain analysis of HRV. The findings of this study may also provide guidance for the medical surveillance of shift working female nurses to help detect early signs of severe sleep disturbances and, in turn, help reduce work accidents and absenteeism, both of which are related to sleep disturbances in nurses who work shifts.

## Figures and Tables

**Table 1:** Demographic characteristics of the participants.

Variables	*n*	%	Mean (SD)
Age			31.48 (7.41)
Nursing seniority			8.95 (6.61)
Education level			
College	376	95.7	
University	17	4.3	
Marital status			
Unmarried	153	38.9	
Married	240	61.1	
Number of children			
None	258	65.6	
1	37	9.4	
2	83	21.1	
Above 3	15	3.8	
Shift work			
No	98	24.9	
Yes	295	75.1	
Time-domain parameters			
Heart rate (bpm)			78.74 ± 11.93
SDNN (ms)			47.71 ± 17.94
RMSSD (ms)			37.97 ± 18.83
Frequency-domain parameters			
TP (ms^2^)			1918.37 ± 1304.90
LF (ms^2^)			844.56 ± 809.36
HF (ms^2^)			529.50 ± 457.42
LF/HF			1.26 ± 1.86

SDNN: standard deviation of normal to normal; RMSSD: root mean square of the successive differences; TP: total power; LF: low-frequency HRV; HF: high-frequency HRV; LF/HF: the ratio of low-frequency power to high-frequency power; SD: standard deviation.

**Table 2:** Mean global and component PSQI scores.

Variables	Mean (SD)	*n* (%)	Range
Subjective sleep quality	1.40 (0.76)		
Very good		33 (8.4)	
Fairly good		202 (51.4)	
Fairly poor		122 (31.0)	
Very poor		36 (9.2)	
Sleep latency (minutes)	1.58 (0.93)		
≤15		53 (13.5)	
16–30		129 (32.8)	
31–60		137 (34.9)	
>60		74 (18.8)	
Sleep duration (hours)	1.56 (1.03)		
>7		67 (17.0)	
>6–7		132 (33.6)	
5–6		98 (24.9)	
<5		96 (24.4)	
Sleep efficiency (%)	2.91 (0.39)		
>85		6 (1.5)	
75~84		11 (2.9)	
<65		376 (95.6)	
Sleep disturbance ^#^	1.32 (0.56)		
Never		11 (2.8)	
Once or twice		253 (64.4)	
Once or twice each week		119 (30.3)	
Three or more times each week		10 (2.5)	
Use of sleep medication	0.27 (0.69)		
Not during the past month		329 (83.7)	
Less than once a week		33 (8.4)	
Once or twice a week		17 (4.3)	
Three or more times a week		14 (3.6)	
Daytime dysfunction	1.22 (0.79)		
0		67 (17.0)	
1–2		193 (49.1)	
3–4		110 (28.0)	
Above 3		23 (5.9)	
PSQI global score	10.20 (2.85)		0–21
Proportion of good sleepers (global score < 5)		16 (4.1)	
Proportion of poor sleepers (global score ≥ 5)		377 (95.9)	

PSQI: Pittsburgh Sleep Quality Index; SD: standard deviation; ^#^ difficulty falling asleep.

**Table 3:** Differences among participants’ demographic variables, HRV parameters, and PSQI global score.

Variables	HRV Parameters							PSQI Global Score
	HR	SDNN	RMSSD	TP	LF	HF	LF/HF	
	Mean (SD)	Mean (SD)	Mean (SD)	Mean (SD)	Mean (SD)	Mean (SD)	Mean (SD)	Mean (SD)
**Education level**								
College	76.88 (12.20)	50.28 (19.08)	41.17 (17.91)	2112.40 (1444.95)	912.57 (940.10)	523.17 (404.04)	1.54 (2.22)	10.19 (2.89)
University	82.06 (10.97)	43.15 (15.13)	32.29 (19.57)	1573.42 (950.66)	723.65 (502.34)	540.74 (552.45)	0.3 (0.57)	10.31 (2.23)
*t* (*p*)	−1.64 (0.692)	1.17 (0.502)	1.37 (0.378)	1.22 (0.491)	1.22 (0.479)	0.36 (0.971)	1.46 (0.430)	−0.27 (0.676)
**Marital status**								
Married	79.50 (12.53)	49.27 (14.89)	36.70 (12.48)	1875.49 (1261.64)	606.19 (499.89)	570.78 (438.30)	1.75 (3.66)	10.11 (2.89)
Unmarried	78.50 (11.89)	47.21 (18.95)	38.37 (20.55)	1931.90 (1334.54)	919.83 (876.92)	516.45 (468.25)	1.10 (0.71)	10.26 (2.82)
*t* (*p*)	0.25 (.803)	0.34 (0.733)	−0.26 (0.792)	−0.12 (0.898)	−1.17 (0.246)	0.35 (0.724)	1.04 (0.558)	−0.48 (0.961)
**Number of children**								
None	78.90 (11.77)	48.16 (18.83)	38.54 (19.99)	1992.42 (1362.31)	902.32 (860.17)	509.08 (451.66)	1.38 (2.02)	10.28 (2.85)
1	87.33 (17.61)	29.15 (6.02)	23.66 (6.85)	817.91 (430.06)	207.44 (114.01)	440.82 (205.35)	0.36 (0.14)	9.73 (2.76)
2	71.20 ± (8.28)	55.18 (6.86)	42.18 (11.91)	2120.93 (1025.59)	782.96 (519.77)	813.85 (604.49)	0.78 (0.69)	10.22 (2.86)
F (*p*)	1.27 (0.295)	1.40 (0.254)	0.66 (0.580)	0.89 (0.475)	0.69 (0.449)	0.86 (0.559)	0.39 (0.467)	0.50 (0.671)
**Shift work**								
Yes	78.98 (12.07)	47.37 (18.21)	37.67 (19.13)	1876.98 (1315.74)	816.85 (814.07)	531.20 (466.97)	1.23 (1.90)	10.24 (2.86)
No	73.00 (7.07)	55.86 (5.44)	45.25 (7.70)	2911.54 (124.17)	1509.48 (171.66)	488.5 (12.41)	1.86 (0.02)	10.00 (2.80)
*t* (*p*)	−0.69 (0.493)	0.65 (0.517)	0.55 (0.582)	4.94 ** (<0.01)	4.10 ** (0.018)	−0.12 (0.899)	0.46 (0.643)	0.71 (0.834)

HR: heart rate; SDNN: standard deviation of normal to normal HRV; RMSSD: root mean square of the successive difference in HRV; TP: total power HRV; LF: low-frequency HRV; HF: high-frequency HRV; LF/HF: low-frequency power to high-frequency power ratio; SD: standard deviation.

**Table 4:** Differences in mean values of HRV parameters between good and poor sleepers.

HRV Parameter	Good Sleepers	Poor Sleepers	*t*	p
	Mean (SD)	Mean (SD)		
Time domain				
Heart rate (bpm)	83.22 (10.92)	77.76 (12.03)	1.25	0.601
SDNN (ms)	49.85 (16.61)	47.24 (18.37)	0.39	0.732
RMSSD (ms)	36.21 (14.62)	38.36 (19.76)	−0.30	0.974
Frequency domain				
LF (ms^2^)	723.39 (666.76)	1396.57 (1171.06)	−2.36	<0.0001
TP (ms^2^)	2975.14 (1652.76)	1686.39 (1110.46)	2.87	<0.0001
HF (ms^2^)	535.44 (491.08)	502.42 (273.41)	0.19	0.209
LF/HF	1.51 (3.27)	0.10 (0.36)	1.72	0.882

SDNN: standard deviation of normal to normal HRV; RMSSD: root mean square of the successive difference in HRV; TP: total power HRV; LF: low-frequency HRV; HF: high-frequency HRV; LF/HF: low-frequency power to high-frequency power ratio. PSQI global score, good sleepers had PSQI < 5 and poor sleepers had PSQI ≥ 5; ms: millisecond; BPM: beats per minute; SD: standard deviation.

**Table 5:** Associations between sleep quality, HRV parameters, and participants’ demographics.

Variables	Age	Nursing Seniority	Heart Rate	SDNN	RMSSD	TP	LF	HF	LF/HF	PSQI Global Score
Age	1									
Nursing seniority	0.921 **	1								
Heart rate	−0.089	−0.120	1							
SDNN	−0.098	−0.102	−0.555 **	1						
RMSSD	−0.103	−0.103	−0.668 **	0.878 **	1					
TP	−0.210	−0.217	−0.338 **	0.790 **	0.653 **	1				
LF	−0.099	−0.088	−0.232	0.445 **	0.302 *	0.717 **	1			
HF	−0.244 *	−0.277 *	−0.377 **	0.570 **	0.633 **	0.593 **	0.180	1		
LF/HF	−0.006	0.017	0.038	0.273 *	0.116	0.372 **	−0.036	−0.149	1	
PSQI global score	0.025	0.024	−0.124	−0.185	−0.075	−0.425 **	−0.269 *	−0.147	−0.266 *	1

* *p* < 0.05; ** *p* < 0.01; SDNN: standard deviation of normal to normal HRV; RMSSD: root mean square of the successive difference in HRV; TP: total power HRV; LF: low-frequency HRV; HF: high-frequency HRV; LF/HF: low-frequency power to high-frequency power ratio; PSQI: Pittsburgh Sleep Quality Index.

**Table 6:** Stepwise multiple regression analysis for quality of sleep and its related factors.

Predictive Factors	B	ß	R	R^2^	*t*	*p*
Constant	11.416				6.550	<0.0001
TP	−0.001	−0.528	0.425	0.164	−3.963	<0.0001
Heart rate	−0.069	−0.302	0.512	0.231	−2.271	0.028

TP: total power HRV.

## Data Availability

Not applicable.
